# MicroRNAs and Oxidative Stress: An Intriguing Crosstalk to Be Exploited in the Management of Type 2 Diabetes

**DOI:** 10.3390/antiox10050802

**Published:** 2021-05-19

**Authors:** Teresa Vezza, Aranzazu M. de Marañón, Francisco Canet, Pedro Díaz-Pozo, Miguel Marti, Pilar D’Ocon, Nadezda Apostolova, Milagros Rocha, Víctor M. Víctor

**Affiliations:** 1 Service of Endocrinology and Nutrition, University Hospital Doctor Peset, Foundation for the Promotion of Health and Biomedical Research in the Valencian Region (FISABIO), 46017 Valencia, Spain; vezza_ter@gva.es (T.V.); amardema@alumni.uv.es (A.M.d.M.); francasu@alumni.uv.es (F.C.); pediazpo@alumni.uv.es (P.D.-P.); 2 Department of Pharmacology, University of Valencia, 46010 Valencia, Spain; miguel.marti@uv.es (M.M.); m.pilar.docon@uv.es (P.D.); nadezda.apostolova@uv.es (N.A.); 3 National Network of Biomedical Research on Hepatic and Digestive Diseases (CIBERehd), 46010 Valencia, Spain; 4 Department of Physiology, University of Valencia, 46010 Valencia, Spain

**Keywords:** microRNA, oxidative stress, redox signaling, type 2 diabetes

## Abstract

Type 2 diabetes is a chronic disease widespread throughout the world, with significant human, social, and economic costs. Its multifactorial etiology leads to persistent hyperglycemia, impaired carbohydrate and fat metabolism, chronic inflammation, and defects in insulin secretion or insulin action, or both. Emerging evidence reveals that oxidative stress has a critical role in the development of type 2 diabetes. Overproduction of reactive oxygen species can promote an imbalance between the production and neutralization of antioxidant defence systems, thus favoring lipid accumulation, cellular stress, and the activation of cytosolic signaling pathways, and inducing β-cell dysfunction, insulin resistance, and tissue inflammation. Over the last few years, microRNAs (miRNAs) have attracted growing attention as important mediators of diverse aspects of oxidative stress. These small endogenous non-coding RNAs of 19–24 nucleotides act as negative regulators of gene expression, including the modulation of redox signaling pathways. The present review aims to provide an overview of the current knowledge concerning the molecular crosstalk that takes place between oxidative stress and microRNAs in the physiopathology of type 2 diabetes, with a special emphasis on its potential as a therapeutic target.

## 1. Introduction

Type 2 diabetes (T2D) is a chronic metabolic disease currently affecting more than 400 million people worldwide, and this number is forecast to exceed 600 million by 2040 [1]. It is characterized by the presence of hyperglycemia and β-cell dysfunction, which lead to a functional deficit and/or early destruction of insulin during its synthesis, inadequate or defective insulin receptor activity, and a series of chronic complications (e.g., cardiovascular diseases, peripheral nerve damage, neuropathy, retinopathy, and nephropathy) [2]. These complications are responsible for the great mortality and morbidity related to the disease.

Growing evidence reveals that oxidative stress plays a critical role in T2D’s pathogenesis and related cardiovascular problems [3,4]. Indeed, the common outcomes of T2D, which include dyslipidemia, insulin resistance (IR), hyperglycemia, and hyperinsulinemia, have been shown to contribute to mitochondrial superoxide overproduction in the myocardium and endothelial cells of small and large vessels. In parallel, these stress stimuli promote different mechanisms, such as accumulation of nitric oxide (NO) and advanced glycation-end products (AGE), increased concentration of cytokines and prostanoids, activation of diacylglycerol (DAG), activation of the polyol-sorbitol pathway, and an increase in protein kinase C (PKC) [5]. These actions, in turn, provoke mitochondrial dysfunction, endoplasmic reticulum (ER) stress, and β-cell apoptosis, further emphasizing the role of oxidative stress in diabetic development and progression [6,7,8].

To date, the management of T2D and its related long-term disorders constitutes a socioeconomic burden and significant public health challenge. Many cases of T2D could be prevented by lifestyle changes, including avoiding alcohol and smoking, maintaining healthy eating habits, and staying physically active [2]. Nevertheless, a substantial proportion of subjects do not adhere to such recommendations, which fuels the need for more effective treatments. In this regard, many drugs that stimulate the secretory activity of β-cells and/or increase insulin target tissues’ sensitivity have been assigned to combating T2D [9]. Unfortunately, in the long-term, patients often abandon their pharmacological medications and require insulin administration. For this reason, novel approaches to the treatment and prevention of this metabolic condition are crucially needed.

Over the last few years, microRNAs (miRNAs, miRs) have become the focus of growing attention as important mediators of diverse aspects of cellular oxidative stress in diabetes. They are endogenous post-transcriptional regulators of 19–24 nucleotides that modulate gene expression and inhibit protein translation by canonically binding to the 3′-untranslated region (UTR) of target genes [10]. However, interaction of miRNAs with gene promoters, coding sequences, and 5′ UTR have also been reported [11]. Of note, emerging reports suggest that miRNAs and oxidative stress influence each other, resulting in a vicious cycle. In particular, oxidative stress seems to regulate the function and biogenesis of several miRNAs, and vice versa, as an imbalance in miRNA expression can lead to oxidative stress by facilitating free radical generation and/or reducing the endogenous antioxidant capacity [12,13,14]. Similarly, miRNAs have been found to regulate pancreatic β-cell function in response to metabolic, oxidative, and inflammatory stress through the modulation of different signaling pathways components [15].

This existing literature points to a molecular crosstalk between microRNAs and oxidative stress in the pathogenesis of T2D and its related health problems. A deeper understanding of the molecular mechanisms influencing this communication could help to design novel therapeutic strategies that enhance the regulation of pancreatic islet insulin action and secretion, or both, under oxidative stress conditions.

## 2. Survey Methodology

A literature search was conducted to provide an update of the current knowledge regarding the molecular crosstalk between oxidative stress and microRNA in the pathophysiology of diabetes and its related complications. PubMed and Google Scholar electronic databases were searched for existing publications on the topic. The key search words used were microRNAs, miRNAs, oxidative stress, redox signaling, and diabetes.

### Study Selection Criteria

All articles fulfilled the following inclusion criteria: (1) reviews and original articles dealing with the molecular crosstalk between oxidative stress and microRNA in the pathogenesis of diabetes and its related disorders; (2) reports of in vivo, in vitro, or human studies; (3) reviews, articles, and original works written in English; and (4) papers published between 2000 and 2021.

## 3. Oxidative Stress and Diabetes

Oxidative stress, defined as elevated intracellular levels of reactive oxygen species (ROS) and decreased in antioxidant content, plays a key role in the onset of diabetes and its related disorders [16]. ROS are short-living, highly bioactive molecules resulting from the reduction of molecular oxygen. They include hydroxyl radical, hydrogen peroxide, superoxide, hypochlorous acid, peroxynitrite, and lipid radicals, among others, all of which serve as secondary messengers in intracellular signaling of different biological processes (cell-cell adhesion and signaling, cell differentiation, development, and cell death).

The main sources of ROS are mitochondria, organelles that host critical metabolic processes such as β-oxidation of fatty acids, adenosine triphosphate (ATP)-generating oxidative phosphorylation (OXPHOS), biosynthetic pathways, and mediation of cell death and calcium chelation, amongst many others [17]. Their primary function is oxidative energy production, which takes place at the electron transport chain (ETC), a sequence of four electron transfer complexes (I–IV) integrated in the mitochondrial inner membrane [18]. This process begins with the appearance of NADH and FADH_2_ as a result of two main routes of energy, the Krebs cycle, and β-oxidation of fatty acids, which shuttle high energy molecules to the ETC. NADH and FADH_2_ interact with ETC complex I and II, respectively. This electron transfer generates a proton gradient between the mitochondrial matrix and the intermembrane space; in turn, the energy accumulated is used by complex V to synthesize ATP. Even though most O_2_ is completely consumed during OXPHOS, a small part (1–2%) becomes superoxide anion (O_2_^−^) at complexes I and III. Under physiological conditions, ROS production and degradation are balanced through antioxidant defence mechanisms, including dismutase (SOD), glutathione peroxidase (GPx), catalase, and the (Keap1)-NRF2-ARE pathway [19]. However, during an inappropriate compensatory response, ROS generation is enhanced considerably in response to different stimuli, thus activating intracellular stress-associated pathways. Notably, accumulating evidence suggests that hyperglycemia, as well as hyperlipidemia, can contribute to low-grade inflammation and oxidative stress, both of which are potentially harmful to healthy cells, since they contribute to central processes that are interrupted during diabetes (insulin secretion, insulin action, or both) [20].

Several pathways have been proposed to explain how hyperglycemia and specifically the metabolic state of diabetes generate damage and oxidative stress in cells and tissues requiring (or not) insulin for efficient glucose uptake. These include autoxidation of glucose, protein kinase C (PKC) dependent activation of NAD(P)H oxidase, increased glycolysis, intercellular activation of the sorbitol (polyol) pathway, an augmented hexosamine pathway, increased intracellular formation of AGEs, and enhanced expression of the receptor for AGEs (RAGE) [21]. The activation of these pathways due to excessive production of ROS and prolonged hyperglycemia contributes to the inhibition of glyceraldehyde-3-phosphate dehydrogenase (GAPDH) and, in turn, to the accumulation of specific precursors of the glycolytic pathway, such as fructose-6-phosphate or glyceraldehyde-3-phosphate. In this regard, the subsequent activation of the polyol pathway causes NADPH depletion, thus contributing to oxidative stress and reducing intracellular levels of glutathione, one of the major intracellular antioxidants [22,23].

It is important to mention that, after prolonged exposure to high glucose levels, other ROS sources are activated, among them AGEs. AGEs are heterogeneous products generated by non-enzymatic glycosylation of lipids or proteins during the hyperglycemic state, and they elicit their function by binding to their receptor, RAGE [24]. This interaction activates the nuclear transcription factor kappa-B (NF-κB) pathway and NADPH oxidase, thus promoting oxidative stress and inflammation, and contributing to diabetic vascular complications [25,26]. In addition, AGEs have been shown to neutralize NO, reduce endothelial NO synthase (eNOS) activity, stimulate cell adhesion molecule expression [27], and increase the potent vasoconstrictor endothelin-1 (ET-1), thus affecting endothelial function and altering the structural integrity of the vascular wall [28]. Growing evidence also suggests that excessively high AGEs are linked to β-cell damage and peripheral IR through a variety of mechanisms, including inhibition of cytochrome-c oxidase and reduction of ATP production [29], impairment of mitochondrial function [30], generation of oxidative stress, and induction of inflammatory events [31]. Regarding the last of these mechanisms, studies performed in human umbilical vein endothelial cells (HUVECs) reveal that AGEs excreted by activated macrophages can increase the expression of proinflammatory mediators such as tumor necrosis factor-α (TNF-α), IL-1β, and IL-6 [24], and further induce mitochondrial dysfunction and cell death [32]. Specifically, AGEs induce the macrophage secretion of HMGB1 and S100, proteins that regulate endothelial cell activation, inflammation, differentiation, proliferation and cell migration, mostly via ERK1/2 and NF-κB activation [33,34]. Accumulating evidence also confirms that AGEs affect insulin secretion as well as insulin gene transcription. For example, Zhao et al. showed that AGEs inhibit ATP production and cytochrome c oxidase activity in murine pancreatic islets, thus inducing iNOS expression and impairing insulin secretion, which leads to an increase in blood glucose concentrations [29]. In this line, Puddu et al. reported that the decreased insulin content observed in the clonal β-cell line HIT-T15 after AGEs treatment is associated with decreased expression of PDX-1 (pancreatic and duodenal homeobox 1) and an increase of FoxO1 (Forkhead box protein O1), both of which play key roles in β-cell maturation, as well as in gluconeogenesis and glycogenolysis. Subsequent research has suggested that AGEs also degrade pancreatic β-cells, thus contributing to their impaired function or apoptosis. Studies by Lim et al. [35] showed that AGEs treatment induces ROS formation and RAGE expression, as well as causing β-cells apoptosis. Antioxidant treatment and RAGE inhibition prevented these changes, suggesting that AGEs stimulate cell death through RAGE-induced ROS generation. Accordingly, Lin et al. reported an apoptotic morphology in AGE-treated INS-1 cells (a well-established model for studies of pancreatic islet beta-cell function). These effects are due to AGE-induced oxidative stress generated through both stress-related signaling pathways (p38 and Jun N-terminal kinase) and the mitochondrial electron transport chain, which activate ROS production via NAPDH oxidase [36]. Although AGEs are principally produced endogenously, they can also be derived from diet (e.g., meat, cheese, coffee, milk), especially from food stored for long periods, prepared under high temperature conditions, or containing additives. Therefore, preventing dietary uptake of AGEs and endogenous AGEs formation may represent an integral part of comprehensive diabetes care.

In recent years, DAG–protein kinase (PK)C activation has received increasing attention as another critical pathway that links hyperglycemia to oxidative stress and the diabetic complications related to it. While DAG is a second-messenger signaling lipid, PKC is a serine/threonine-related protein kinase associated with vascular alterations such as angiogenesis, permeability, defects in extracellular matrix synthesis, cytokine activation and inhibition, cell growth and apoptosis, and leukocyte adhesion [37]. It is widely accepted that high glucose levels activate the formation of DAG, which, in turn, binds to PKC and causes its activation and translocation to the plasma membranes. PKC phosphorylates NADPH oxidase, thereby stimulating, either directly or indirectly, the generation of superoxide and further promoting oxidative stress [38]. Consequently, activation of the DAG/PKC pathway leads to phosphorylation and reduced activity of eNOS, along with an increment of platelet aggregation and vasoconstriction via ET-1 production. In addition, this pathway has been shown to contribute to the inflammatory process by activating adhesion molecules and cytokines [21,38,39]. Interestingly, PKC activation has also been shown to inhibit downstream metabolic enzymes and different insulin signaling cascade components [40]. Indeed, PKC seems to directly phosphorylate serine residues of the insulin receptor substrates, especially IRS-1, and promote their degradation, thus attenuating insulin signaling and inducing IR [41].

Of note, pancreatic β-cells are particularly vulnerable to oxidative stress, as they express relatively low levels of some antioxidant systems and peroxide-metabolizing enzymes (i.e., GSH peroxidase, catalase, and SOD). Under physiological conditions, β-cells release insulin in response to blood glucose levels. However, conditions like hyperglycemia, increased metabolic stress, and peripheral IR can lead to mitochondrial dysfunction, activation of the mitochondrial cytochrome c-mediated apoptotic pathway, and enhanced ROS generation. The oxidative stress generated can directly damage β-cells by oxidizing lipids, proteins, and DNA, which causes their dysfunction and/or death through several processes such as changes in dysregulated gene expression, receptor signal transduction, enzymatic activity, ion channel transport, and apoptosis [42,43]. Consequently, both insulin action and production by β-cells become deficient.

Excessive ROS levels can also indirectly damage β-cells by activating different stress-sensitive intracellular signaling pathway mediators such as p38 mitogen-activated protein kinases (p38 MAPK), NF-κB, c-Jun N-terminal kinase/stress activated protein kinases (JNK/SAPK), among others. These changes can reduce mitochondrial ATP production by down-regulating respiratory chain proteins, thus impairing insulin production [44]. Several mitochondrial pathways are reported to be implicated in ROS production in β-cells under hyperglycemia, including increased intracellular AGE production, oxidative phosphorylation, protein kinase C (PKC) activation, and polyol pathway activation, which have been mentioned previously.

Regarding pancreatic β-cell function and survival, oxidative stress interferes with three essential pathways: c-Jun N-terminal kinase (JNK) activation, AMP-activated protein kinase (AMPK) activation, and mammalian target of rapamycin (mTOR) inhibition. The AMPK pathway regulates several β-cell processes, including proliferation, insulin secretion, and survival. Under normal conditions, glucose stimulation leads to a reduction in AMPK phosphorylation and related activation; however, this reduction is markedly attenuated in pathological states [45]. In vivo studies performed by Zhang et al. demonstrated that ROS-mediated overexpression of pAMPK increases extracellular-signal-regulated kinase (pERK), which is involved in reduced β-cell mass and impaired β-cell proliferation [46]. Moreover, pAMPK up-regulation increased β-cell death and reduced insulin production in murine pancreatic cells. Interestingly, pAMPK may have an inhibitory effect on mTOR, a nutrient-responsive serine-threonine kinase that plays a crucial role in increasing and maintaining β-cell mass by regulating autophagy, cell growth, translation, cell size, apoptosis, and proliferation [47]. Indeed, it is known that, under oxidative stress, mTOR is inhibited through the activation of AMPK. Thus, mTOR inactivation leads to several detrimental effects in multiple downstream intracellular processes, among which increased expression of the thioredoxin-interacting protein (TXNIP) is the most relevant [48,49]. TXNIP is a ubiquitously expressed protein that negatively modulates the TXN antioxidant systems, influencing cellular antiapoptotic and antioxidant mechanisms [50]. In this regard, several studies have confirmed that, once inside the mitochondria, TXNIP binds to TXN2 and initiates mitochondria-mediated β-cell apoptosis through the apoptosis-signal-regulating kinase, namely ASK1 [51]. Of note, TXNIP-induced apoptosis is associated with activation of the NLR family pyrin domain containing 3 (NLRP3) inflammasome, a multimeric protein complex known to influence the innate immune system. Particularly, TXNIP induces NLRP3 inflammasome assembly, which recruits procaspase-1 to generate active caspase-1 and then converts the immature cytokines pro-IL-18 and pro-IL-1β into their mature forms, IL-18 and IL-1β. These activated cytokines contribute to the subsequent inflammatory effect, thus mediating oxidative stress-induced pancreatic islet dysfunction [52].

Another essential pathway activated in β-cells under oxidative stress conditions is the JNK pathway. JNK activation is involved in promoting impaired insulin signaling and apoptosis through serine phosphorylation and further inactivation of insulin receptor substrate 1/2 (IRS1/2) and the phosphoinositide 3-kinase (PI3K)/protein kinase B (AKT) pathway [53,54]. Concurrently, inactivation of the PI3K/AKT pathway leads to down-regulation of mTOR and the subsequent loss of β-cell mass, as well as the nuclear translocation of FOXO1 and reduction of PDX-1 [54], which ultimately stunts β-cell growth and proliferation [55].

**Figure 1:**
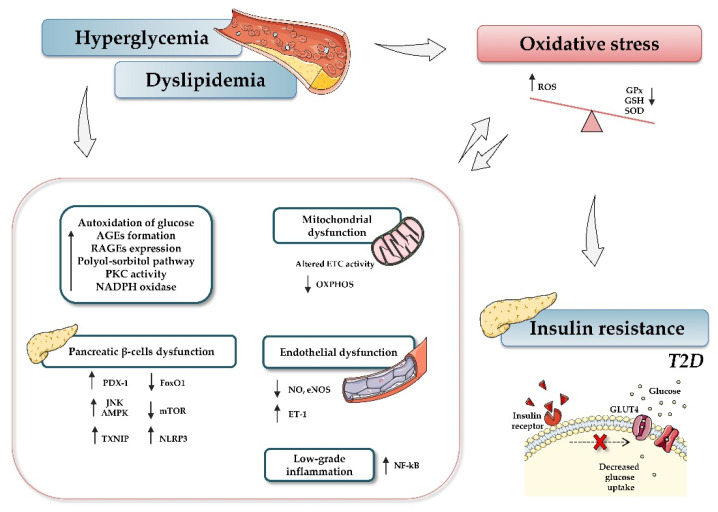
Relationship between type 2 diabetes and oxidative stress. Hyperglycemia, dyslipidemia, and insulin resistance play key roles in oxidative stress and diabetes development. Briefly, increased glucose levels promote different mechanisms, such as autoxidation of glucose, accumulation of advanced glycation-end products (AGE) and nitric oxide (NO), activation of diacylglycerol (DAG), activation of polyol-sorbitol pathway, and an increase in protein kinase C (PKC), which, in turn, lead to the generation of oxidative stress, impairment of mitochondrial function, and induction of inflammatory events. The figure summarizes the most relevant involved processes and signaling pathways. Up and down arrows indicate an increase and decrease, respectively. Abbreviations: AGEs, advanced glycation-end products; AMPK, AMP-activated protein kinase; ETC, electron transport chain; FoxO1, Forkhead box protein O1; GPx, glutathione peroxidase; GSH, reduced glutathione; NADPH, nicotinamide adenine dinucleotide phosphate; NF-кB, nuclear factor kappa-light-chain-enhancer of activated B cells; NO, nitric oxide; eNOS, endothelial nitric oxide synthase; mTOR, mammalian target of rapamycin; NLRP3, (nucleotide oligomerization domain (NOD), leucine-rich repeat (LRR) and pyrin domain (PYD)); OXPHOS, oxidative phosphorylation system; PDX-1, pancreatic and duodenal homeobox 1; PKC, protein kinase C; RAGEs, receptor for AGEs; ROS, reactive oxygen species; SOD, dismutase; JNK, c-Jun N-terminal kinase; T2D, type 2 diabetes; TXNIP, thioredoxin-interacting protein.

In light of the reported knowledge, hyperglycemia and oxidative stress appear to be involved in different signaling pathways that contribute to β-cell dysfunction, tissue inflammation, and insulin resistance (Figure 1). Therefore, preservation of glucose and redox homeostasis is essential to prevent diabetes. In this regard, microRNAs (miRNAs, miRs) have become the focus of increasing interest as important mediators in regulating diverse aspects of the cellular oxidative stress typical of diabetic conditions.

## 4. MiRNAs

**Figure 2:**
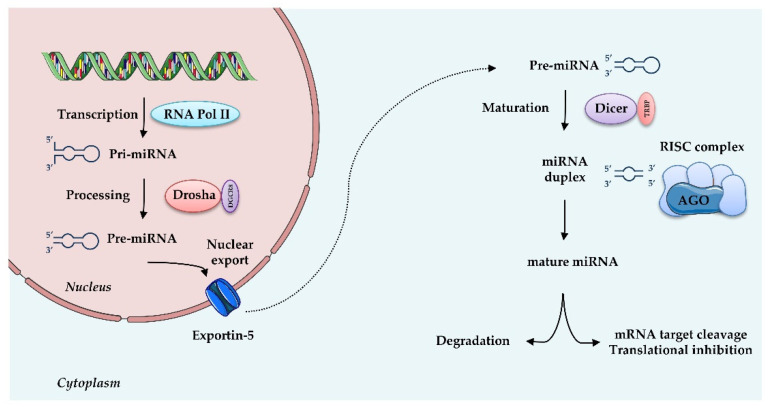
MicroRNAs biogenesis: microRNAs are transcribed by RNA Polymerase II (RNA Pol II) as large RNA precursors named pri-miRNA. Pri-miRNA is processed by the enzyme Drosha in association with the RNA-binding protein DGCR8 (DiGeorge syndrome critical region gene 8), which excises the stem-loop to form pre-miRNA. Pre-miRNAs are exported from the nucleus by a carrier protein known as Exportin-5. In the cytoplasm, pre-miRNA undergoes further processing by the Dicer-TRBP complex into a miRNA duplex approximately 20 nt long, known as 3′ and 5′ end strands. Typically, the first strand is degraded and the second is selected as mature miRNA. Mature miRNA is loaded into RNA induced silencing complex (RISC) via the AGO proteins and is then able to regulate gene expression by translational repression or induction of mRNA degradation. Increasing evidence suggests that, depending on the cell type or tissue, both strands can generate functional mature miRNAs.

MiRNAs are small (20–25 nt) non-coding, single stranded RNAs that inhibit gene expression at the transcriptional or post-transcriptional level. They are known to target multiple genes, and, conversely, genes are targeted by multiple miRNAs, thus allowing many levels of regulation of gene expression. MiRNAs originate from a double-stranded RNA transcript folded in a hairpin structure, known as pre-miRNA precursor molecules. The canonical biogenesis of miRNAs requires two central steps; in the nucleus, the enzyme Drosha, in association with the double-stranded RNA-binding protein DGCR8, crops pre-miRNA to a 70-nucleotide (nt) pre-miRNA, which is then transported to the cytosol. There, pre-miRNA undergoes further processing by the Dicer-TRBP (transactivation-responsive RNA-binding protein) complex into a miRNA duplex approximately 20 nt long, with a 3′- strand and a 5′- terminus strand. Although both arms can become functional, one strand is typically degraded and the other is selected as mature miRNA, depending on the cell type or the tissue. Subsequently, this single-stranded miRNA directly interacts with proteins of the Argonaute (AGO) family, components of “miRNA-induced silencing complexes” (miRISCs). These effector complexes promote mRNA deadenylation, translational repression or endonucleolytic cleavage of highly complementary targets, and exonucleolytic decay of partially complementary ones [56]. Besides this canonical miRNA biogenesis, illustrated in Figure 2, an alternative non-canonical pathway that bypasses Drosha or Dicer processing has been described [57]. miRISC, through sequence-specific binding to the UTR of target mRNAs, is able to guide the miRNA to the specific target, thus interfering with translational initiation factors and translation and protein synthesis, as well as blocking translation post-initiation [57].

It is thought that miRNAs modulate the expression of up to 60% of the human genome’s genes [58], and many of them have been shown to participate in the development of diabetes. Particularly, several dysregulated miRNAs from insulin-sensitive organs, including insulin-producing pancreatic β-cells, white adipose tissue, and skeletal muscle, have been related to various diabetes-associated processes, including insulin secretion, endothelial dysfunction, adipocyte differentiation, and pancreatic β-cells [59,60].

## 5. Oxidative Stress, miRNAs, and Diabetes

This oxidative stress has been implicated in several pathological mechanisms of diabetes: deregulation of antioxidant pathways, inflammation, altered glycemic control, changes in lipid metabolism and structure, protein oxidation, and mitochondrial and β-cell dysfunction. Therapeutic approaches aimed at reducing oxidative stress may offer protection against diabetes and its complications. However, antioxidant treatments have mostly proved unsuccessful to date [61]. In recent years, many studies have pointed to a key role for several miRNAs in oxidative stress (Table 1).

Consequently, the restoration of miRNA expression to normal levels may represent an alternative therapeutic intervention to counteract oxidative damage.

Multiple miRNAs have been evaluated in cellular and animal models of diabetes, as well as in human samples, and we aim to provide an overview of the latest and most important ones.

### 5.1. MiRNAs, Hyperglycemia-Induced ROS and Endothelial Dysfunction

A recent report has shown that patients with T2D and impaired glucose tolerance present increased miR-21 plasma levels [82], which leads to an undermined antioxidant response and disruption of ROS homeostasis. Indeed, miRNA-21 inhibition was able to reverse the effect of its putative ROS-homeostatic target genes, such as FoxO1 and SOD2 [83], whose expression is diminished under diabetic conditions. These findings were confirmed by an in vivo experiment in mice in which miR-21 suppression stimulated the nuclear peroxisome proliferator activated receptor (PPAR) [84], known to be an important receptor in the regulation of homeostasis, and the metabolism of glucose and lipids [85]. In this context, many studies have demonstrated the crucial role of the miR-200 family in the oxidative stress and endothelial inflammation present in diabetes and its associated diseases [63]. The miR-200 family includes different evolutionarily conserved miRNAs (miR-429, miR-141, miR-200a, miR-200b, and miR-200c) implicated in the regulation of redox imbalance and positively modulated by H_2_O_2_. In particular, it has been found that overexpression of miR-200c reduces endothelial cell growth in HUVECs, thus inducing their senescence and apoptosis, while inhibition of the same miRNA leads to the restoration of endothelial function [63]. The endothelial function has been seen to take place via up-regulation of zinc finger E-box-binding homeobox (ZEB1) [63], a homeodomain transcription factor that protects against oxidative stress-associated endothelial dysfunction. Similarly, Belgardt et al. revealed that mice treated with streptozotocin (STZ), an agent that causes DNA damage, oxidative stress, and subsequently diabetes, displayed overexpression of miR-200 in pancreatic β-cells. In contrast, miR-200–silenced mice exhibited unaltered β-cell function and normal metabolic parameters, and were partially protected from STZ-induced hyperglycemia [86]. Interestingly, in vitro findings have demonstrated that miR-200 can also regulate sirtuin 1 (SIRT1) [62], a class III histone deacetylase that acts as a sensor of oxidative stress and a modulator of several cellular mechanisms, including insulin resistance and energy balance. Generally, SIRT-1 cooperates with AMPK in order to activate FOXOs, peroxisome proliferator-activated receptor γ coactivator-1α (PGC-1α) and PPARα, and, in turn, antioxidant signals, to suppress stress. By targeting SIRT1, miR-200 decreases NO and increases the acetylation of SIRT1 targets, thus causing ROS generation and endothelial dysfunction. Subsequent in vivo studies have confirmed these data, highlighting that elevated ROS production occurs through the targeting and negative regulation of peroxiredoxin 2 (PRDX2) [62], a redox protein that contributes to the antioxidant defence system. Of note, SIRT1 is targeted by other miRNAs, thus increasing diabetes-associated oxidative stress. Among these miRNAs, miR-34, miR-204, and miR-106b can be counted [87]. MiR-34 downregulates SIRT1, causing hyperglycemia-induced vascular cell senescence [65]; miR-204 promotes endothelial dysfunction and vascular ER stress [88]; and miR-106b overexpression increases the oxidative stress induced by high glucose levels in NIT-1 cells, a mouse pancreatic β-cell line [66].

### 5.2. MiRNAs and AGE-Induced Oxidative Stress

As previously mentioned, prolonged exposure to high glucose levels can generate AGEs, which activate several signaling pathways that elicit ROS production, NO activity, and chronic and acute diabetes-related inflammatory processes. In this line, several miRNAs are reported to play an essential role in AGE modulation. Li et al. [67] observed significant up-regulation of miR-214 in human AGE-treated THP-1 monocytes, which delayed their apoptotic processes and acted as a molecular signature of chronic inflammation. A similar up-regulation of miR-214 was also detected in peripheral monocytes from patients with chronic renal failure. Interestingly, using bioinformatics analysis, the authors provided a list of predicted miR-214 target genes, among which the most relevant was PTEN, a pro-apoptotic molecule that normally acts on AKT [89]. Upon overexpression of miR-214, the amount of PTEN decreases and is unable to activate Akt, thus contributing to monocyte growth and survival and the inflammation that subsequently occurs [67]. In addition, studies performed in human renal tubular cells have indicated that AGE-induced oxidative and ER stress reduces miR-205 and induces ROS generation by reducing heme oxygenase-1 (HO-1), SOD-1 and SOD-2 [68]. These findings suggest that miR-205 improves antioxidant enzyme expression, and that its down-regulation results in the impairment of cell survival under stress. Moreover, up-regulation of miR-21 has been related to oxidative stress in that it reduces NO bioavailability and increases intracellular ROS levels via SOD2 targeting [69]. Of special relevance to the AGE-induced oxidative stress axis is miR-200b/miR-200c. Loss of miR-200b/miR-200c function in AGE-treated endothelial cells has been shown to induce ROS production and apoptosis via up-regulation of RhoA/RhoA/Rho associated kinase 2 (ROCK2) signaling [64]. ROCK is a serine/threonine kinase that interacts with activated Rho GTPases, thus controlling several critical cell processes, such as proliferation, differentiation, motility, adhesion, apoptosis, and ROS generation. Wu et al. provided evidence that the ROCK signaling pathway is an essential player in oxidative stress-induced damage in HUVECs. The increased cell permeability they observed was partially due to F-actin depolymerization in response to AGEs. Reduction of RhoA/ROCK2 signaling by miR-200c and miR-200b mimics led to F-actin remodeling and a further amelioration of AGE-induced injury. Unfortunately, the exact mechanism responsible for this is unclear, and further studies are warranted to clarify it.

### 5.3. MiRNAs and Pancreatic β-Cell Function

Accumulating evidence also suggests that miRNAs are key players in pancreas β-cell differentiation and function. Among them, miR-375, a pancreatic islet-specific miRNA, has been involved in the final stages of insulin secretion by regulating myotrophin expression in cultured MIN6 cells [70], a pancreatic β-cell line. Myotrophin is a cytoplasmic protein involved in insulin granule exocytosis. Consequently, miR-375 overproduction and the reduced expression of its target myotrophin attenuate insulin release [70]. Based on these data, further murine studies were performed, revealing that the specific knockdown of miR-375 led to reduced β-cell mass and proliferation, increased glucagon release from isolated islets, and subsequent hepatic gluconeogenesis and glucose production [71]. The process by which loss of miR-375 function directs to reduced cell mass is currently unknown and requires further research.

Interestingly, many researchers underline the important role of miRNAs in controlling β-cell insulin content through the downregulation of several transcriptional repressors. Melkman-Zehava et al. demonstrated that specific suppression of miR-182, miR-148, miR-26, or miR-24 in isolated primary islets or cultured MIN6 cells downregulates insulin mRNA levels and insulin promoter activity [72]. Indeed, knockdown of the above mentioned miRNAs has been shown to significantly upregulate Sox6 and Bhlhe22, two target genes known to repress insulin expression [90].

Similar to miR-24, miR-26, miR-148, and miR-182, miR-30d is a positive regulator of insulin transcription. Specifically, it downregulates mitogen-activated protein 4 kinase 4 (MAP4K4), a cytokine-inducible kinase that, when activated, inhibits insulin production and release [73]. In this regard, a reduction of miR-30d has been detected in islets isolated from diabetic mice, in which the expression of MAP4K4 was markedly augmented. The same study revealed that miR-30d induces insulin gene transcription by activating MafA (v-maf musculoaponeurotic fibrosarcoma oncogene homolog A), a transcription factor that regulates β-cell activity, affecting β-cell mass, insulin transcription, and insulin secretion. Several reports have indicated that the sustained expression of MafA results in augmented β-cell mass, higher plasma insulin levels, and significantly lower plasma glucose levels [74], thus painting a picture of miR-30d as a promising therapeutic target for diabetes management.

Of particular relevance in this sense is miR-7, a negative regulator of insulin secretion in β-cells. Latreille et al. showed that miR-7 regulates β-cell function by directly targeting genes that regulate glucose-stimulated insulin secretion [75], and its genetic deletion improves glucose tolerance in mice. Similarly, using transgenic mice, they demonstrated that overexpression of miR-7 leads to β-cell dedifferentiation, impaired insulin release and, subsequently, diabetes [75]. Lastly, the same authors analyzed miR-7 levels in islets from mildly T2D, non-diabetic obese, and control human subjects. As expected, miR-7 levels were lower in non-diabetic and obese individuals, thus confirming the results obtained in mouse models. Surprisingly, T2D patient islets also displayed lower miR-7 levels than healthy controls, thus denoting a transient phase of the disease immediately prior to a prediabetes condition [75].

In the last few years, miR-7a, the most abundant form of miR-7 in islet cells, has been shown to inhibit mTORC signaling, an intracellular pathway that coordinates insulin signaling by regulating different components, such as insulin-like growth factor 1 receptor/insulin receptor (IGF-IR/IR), insulin receptor substrate (IRS-1), growth factor receptor-bound protein 10 (Grb10), and F-box/WD repeat-containing protein 8 (Fbw8), among others [91]. MiR-7 seems to act on five of its main components: two MAPK-interacting kinases, Mknk1 and Mknk2; two main downstream effectors of eukaryotic translation initiation factor 4E (eIF4E), p70S6K and TORC1; and one of the essential TORC2 components, Mapkap1. Wang et al. also demonstrated that silencing miR-7a in mouse primary islets can activate mTOR signaling and promote β-cell replication and proliferation [92]. For this reason, miR-7 is one of the most extensively evaluated RNA regulators and potentially attractive therapeutic targets in diabetes, since it represents a “brake” for β-cell proliferation.

Like miR-7, miR-9 exerts a negative control on glucose-induced insulin release in β-cells. In this regard, Roggli et al. suggested that overexpression of miR-9 in MIN6 and dissociated islet cells is responsible for defective insulin release by directly targeting the transcription factor Onecut2. This factor is known to bind to the granuphilin promoter, a potent inhibitor of insulin exocytosis [76], and to repress its expression and transcriptional activity, thus undermining the secretion of insulin. In addition, Roggli et al. demonstrated that overexpression of Onecut2 alters insulin secretion in INS-1E cells, a widely used β-cell model, suggesting that excessive levels of Onecut2 can be deleterious for β-cell function.

### 5.4. MitomiR

As reported in some of our previous reviews and original studies [7,8], mitochondrial dysfunction associated with oxidation of fatty acids is closely linked to oxidative stress and the further onset of insulin resistance and T2D. Notably, some recently discovered miRNAs, known as mitochondrial-located miRNAs (MitomiR), have been shown to directly alter mitochondrial functions. In this sense, they seem to interact with mitochondrial genome-derived mRNA molecules and further regulate several proteins that are critical for these organelles’ metabolism, as well as for OXPHOS and lipid metabolism [93,94]. Studies performed in skeletal muscle from transgenic mice showed that overexpression of miR-23a leads to a reduction in PGC-1α and undermined mitochondrial function and biogenesis. Similarly, Junaith et al. reported that high fat diet (HFD)-induced mitochondrial dysfunction in skeletal muscle is associated with miR-149 down-regulation, which interferes with the SIRT-1/PGC-1α pathway [77]. The authors demonstrated that miR-149 inhibits poly(ADP-ribose) polymerase-2 (PARP-2) and increases cellular NAD+ levels and SIRT-1 activity, which further enhances mitochondrial biogenesis and function through PGC-1α activation. Indeed, it is known that some miRNAs modulate mitochondrial energy metabolism and OXPHOS by targeting various of the main mitochondrial components implicated in ETC, ATP production, and electron transfer; for example, miR-141 downregulates complex V in the mitochondrial ETC; miR-338, miR-210, and miR-181c, downregulate complex IV; and miR-210 downregulates complex III [80,81].

In light of the above-mentioned information, oxidative stress seems to be crucial for the regulation and deregulation of miRNA networks, and vice versa, as miRNA acts as a main player in different physiological and pathological events, making both of them attractive therapeutic targets.

## 6. MiRNAs, Therapeutic Targets, and Delivery Systems

Thanks to their essential ability to target multiple genes, miRNAs are considered upstream regulators of multiple biological processes. Therefore, the direct targeting of a single miRNA (or a set of miRNAs) in specific tissues or cell types may drive the modulation and regulation of diverse physiological and pathological pathways. For this reason, various therapeutic approaches based on miRNA modulation have gained increasing attention from researchers working in diabetic healthcare management. As already mentioned, miRNAs can control the expression of different factors that prevent or cause oxidative stress in diabetes and its associated pathological disorders. Various strategies and approaches to restore the dysregulated expression (overexpression or underexpression) of miRNAs involved in diabetes have recently been developed. In these strategies, known as “miRNA mimicking or inhibition”, non-natural or artificial small RNA bind specifically to their target sequence, thus producing a translational replacement or inhibition of the gene [95]. They include miRNA sponges and antisense oligonucleotides. While miRNA sponges are double-stranded transcripts that exist in both linear and circular forms, and which bind to high-specific miRNAs and competitively sequester them from their natural targets, antisense oligonucleotides are single-strand RNA molecules that bind to the target miRNA and inhibit or degrade its function. However, both mimicking and inhibition have been shown to have their limitations: they lack a reliable and safe strategy to target specific cells, tissues, and organs, and are naturally susceptible to degradation by exonucleases and endonucleases [96]. In addition to insufficient therapeutic efficacy, there have been reports of immune-related and dose-limiting toxicity and of off-target effects with miRNA mimicking and inhibition strategies. In relation to the second problem, a miRNA-like effect (due to the short sequence complementarity) and saturation of the RNA machinery (due to an overload of miRNAs and a subsequent incorrect guide strand selection by the RISC or other molecules involved in the pathway) are the most common off-target effects described [97]. These undesirable effects are mainly due to the large number of miRNAs and imperfect base-matching with the complementary sites of their targets. Therefore, reducing and circumventing these problems by means of target prediction approaches are research priorities in the development of miRNA therapeutics. To date, several off-target computational algorithms (e.g., Sylamer, DIANA-microT, mirTarget) have been designed and made available to researchers in order to correct UTR length and compositional biases [98,99]. However, further exploration of possible alternative strategies is needed.

In parallel, new technologies are currently being investigated, developed, and employed as potential highly efficient delivery systems aimed at modulating miRNAs, among which inorganic nanoparticles (NPs), organic NPs, and aptamers are the most prominent [100]. Inorganic NPs, composed of cerium oxide, iron oxide, mesoporous silicon, or gold, are biocompatible, hydrophilic, non-toxic, and highly stable particles. Indeed, studies performed in diabetic mice have shown that the administration of anti-miR-155 gold nanoparticles helps to restore cardiac function [101]. Similarly, nanoparticle–microRNA-146a-5p polyplexes have been demonstrated to ameliorate diabetic peripheral neuropathy in rats by regulating apoptosis and expression of inflammatory cytokines [102]. However, despite their optimal properties, these nanomaterials provoke critical alterations in cellular responses to oxidative stress and ROS generation, and consequently should be ruled out as a therapeutic method to decrease oxidative stress [103]. In this line, organic NPs possess properties that make them ideal for delivering miRNA; namely, a low immunological rejection rate and more prolonged survival in blood. They include liposomes, cationic lipid bilayers, and dendrimers, symmetrical hyperbranched polymers whose nucleic acids easily bind through their phosphate-generated negative charge [104,105]. Surface chemical modifications of liposomes and dendrimers can also extend their biocompatibility, enhance targeted specificity and circulation time, and reduce inactivation or degradation by RNAses [100].

Regarding these organic NPs, dendrimers would seem to be the most attractive delivery systems aimed at modulating miRNAs. Studies performed by Akhtar et al. in diabetic rats showed that daily administration of nano-sized polyamidoamine (PAMAM) dendrimers have positive effects on vascular dysfunction [106]. In particular, PAMAM dendrimers were able to inhibit the epidermal growth factor receptor (EGFR)-ERK1/2-ROCK pathway, a key player in the development of vascular abnormalities in diabetes [107]. Similarly, studies performed in HUVECs reported that a dendrimeric “bowtie”, of which one-half is devoted to ligand presentation and the other half to miRNA binding, successfully downregulates the targeted regulating gene of the MAP kinase cascade SPRED1 mRNA with miR-126 [108]. Interestingly, the hyperbranched architecture of dendrimers can increase the delivery of therapeutic RNA thanks to their augmented carrying capacity. These branches are also constituted by a large number of functional terminal groups that are capable of binding many molecules efficiently. Of note, and in contrast to other polymers, dendrimers also have a well-established size after synthesis [109].

Last, but not least, growing evidence endorses the efficacy of miRNA inhibitors or mimics conjugated to a three-dimensional structure, known as an aptamer [110]. Aptamers are short single-stranded oligonucleotides of 15 to 70 nucleotides that are selected for binding to a specific target, which can be peptides, whole cells, proteins, small molecules, toxins, or tissue [111]. They bind selectively to their target through van der Waals forces, hydrogen bonding, base stacking, electrostatic interactions, or a combination of these.

Researchers, among them Trajkovski et al. [112], have demonstrated the therapeutic silencing effect of miRNAs in a murine model of obesity/T2D. The aforementioned authors administered modified anti-miRNA oligonucleotides to HFD-C57BL/6J and *ob/ob* mice by tail-vein injection to suppress both miR-107 and miR103 in the adipose tissue and liver. The results revealed that the silencing of miR-107 and miR-103, which are elevated in the livers of obese/diabetic mice and humans, markedly enhanced hyperglycemia by facilitating insulin sensitivity in the adipose tissue and liver.

Similarly, Choi et al. [113] showed that silencing miR-34a in obese mice using modified anti-miRNA oligonucleotides led to significant amelioration of insulin sensitivity and glucose tolerance, allowing levels obtained in lean control mice to be reached. Moreover, aptamer-mediated suppression of miR-802, a miRNA implicated in the development of IR, has been shown to improve glucose tolerance in the liver of obese and diabetic animals [114].

Despite novel and exciting advances in the development of potent miRNA-silencing strategies, there remain several major challenges to their clinical application—among them, the lack of efficient delivery systems with low toxicity—and, so, further, more thorough investigation is vital.

## 7. Conclusions

Type 2 diabetes is a complex and heterogeneous disease affecting millions throughout the world. Due to increasing trends toward a sedentary lifestyle and obesity, the number of affected subjects is increasing at an alarming rate and is expected to double within the next 10 years. Hyperglycemia, altered insulin secretion, and insulin-target tissue resistance are the primary causes, and are all induced mainly by mechanisms activated by, or as a consequence of, oxidative stress. Recently, miRNA dysfunction and oxidative stress have been shown to be closely linked to the development of diabetes. miRNAs are small regulatory molecules with well-defined physicochemical properties that modulate redox signaling pathways and alter their functionality, stability, and integrity. Conversely, oxidative stress affects the expression levels of multiple microRNAs. Based on this inextricable intertwining, miRNAs represent promising therapeutic targets for diabetes therapy, since they can be easily modified to ameliorate their stability and promote their tissular and cellular delivery, by which complications due to ROS production can be reduced.

Although the emerging knowledge about the therapeutic potential of miRNA modulation in diabetes is encouraging, the underlying mechanisms of action remain an enigma. Important questions in this regard await answers; for example, “What are the underlying molecular mechanisms of miRNAs in the control of oxidative stress in pancreatic β-cell function? Can the manipulation of these regulatory molecules lead to undesirable and drastic effects? Is their effect transient? Will chronic treatments require repeated injections that may lead to safety concerns and untenable costs, among other problems?”

Despite real advances made in the design of novel drugs, further research to enable a better understanding of miRNA as a targeted strategy could revolutionize and improve the management of diabetic patients.

## Figures and Tables

**Table 1:** MiRNAs implicated in type 2 diabetes and its oxidative stress-induced complications. Consequently, the restoration of miRNA expression to normal levels may represent an alternative therapeutic intervention to counteract oxidative damage.

miRNA	Up/DownRegulation	Cell Type/Tissue	Target Gene	Effects	Reference
miR-200 family members	Up-regulation	HUVECs; in vivo mouse pancreatic β-cells	SIRT1 PRDX2	Reduction of endothelial cell growth; elevated ROS production and apoptosis	[62]
miR-200b/miR-200c	Down-regulation	HUVECs	ROCK2	Elevated ROS production and apoptosis	[63,64]
miR-34	Up-regulation	Type 2 diabetic *db/db* mice	SIRT1	Vascular cells senescence	[65]
miR-106b	Up-regulation	Mouse pancreatic β-cell line NIT-1 cells	SIRT1	Increased oxidative stress	[66]
miR-204	Up-regulation	HUVECs; C57/Bl6 mice	SIRT1 CHOP ATF6	Endothelial dysfunction and vascular endoplasmic reticulum stress	[65]
miR-214	Down-regulation	THP-1 cells	PTEN	Apoptosis and development of inflammatory responses	[67]
miR-205	Down-regulation	Human renal tubular HK-2 cells	EGLN2	Elevated ROS production and suppression of antioxidant enzymes	[68]
miR-21	Up-regulation	Human APCs	FoxO1 SOD2	Reduction of NO bioavailability and increased intracellular ROS levels	[69]
miR-375	Down-regulation	MIN6 cells; INS-1E cells; miR-375^−^/^−^, Lep^−^/^−^ (375/*ob*) mice; diabetic GK rats	Aifm1 Pdk1 MTPN	Attenuation of insulin release; reduction of β-cell mass and proliferation	[70,71]
miR-182 miR-148 miR-2 6miR-24	Down-regulation	MIN6 cells; isolated primary islets	Sox 6Bhlhe22	Down-regulation of insulin mRNA levels and insulin promoter activity	[72]
miR-30d	Down-regulation	Islets isolated from *db/db* mice	MAP4K4 MafA	Inhibition of insulin production and release	[73,74]
miR-7	Up-regulation	Transgenic mice; human islets from mildly T2D, obese non-diabetic, and control subjects	mTOR	β-cell differentiation; impaired insulin release	[75]
miR-9	Up-regulation	MIN6 and dissociated islet cells	Onecut 2	Inhibition of glucose-stimulated insulin exocytosis	[76]
miR-23a					
miR-149	Down-regulation	Transgenic mice; skeletal muscle from HFD-fed obese mice	SIRT-1 PGC-1α	Altered mitochondrial function and biogenesis	[77]
miR-141	Down-regulation	Cardiac myocytes from diabetic mice	Slc25a3	Alteration of mitochondrial function (ETC-complex V)	[78]
miR-338 miR-210 miR-181c	Down-regulation	Cardiac myocytes from diabetic rats	COX1 COXIV	Alteration of mitochondrial function (ETC-complex IV)	[79,80]
miR-210	Down-regulation	H9c2 cardiomyocytes	ISCU COX10	Alteration of mitochondrial function (ETC-complex III) and up-regulation of glycolysis	[81]

Abbreviations: Aifm1, apoptosis inducing factor mitochondria associated 1; APCs, angiogenic progenitor cells; ATF6, activating transcription factor 6; Bhlhe22, Basic Helix-Loop-Helix Family Member E22; CHOP, C/EBP homologous protein; COX, cytochrome c oxidase; EGLN2, Egl nine homolog 2; ETC, electron transport chain; FoxO1, forkhead box protein O1; HUVECs, human umbilical vein endothelial cells; HO-1, heme oxygenase-1; IR, insulin resistance; ISCU, iron-sulfur cluster assembly enzyme; MafA, v-maf musculoaponeurotic fibrosarcoma oncogene homolog A; MAP4K4, mitogen-activated protein kinase kinase kinase kinase 4; MTPN, myotrophin; mTOR, mammalian target of rapamycin; Pdk1, phosphoinositide 3-kinase-dependent-kinase; PGC-1α, peroxisome proliferator–activated receptor γ coactivator-1α; PRDX2, peroxiredoxin 2; PTEN, phosphatase and tensin homolog; ROCK2, RhoA/RhoA/Rho associated kinase 2; Slc25a3, solute carrier family 25 member 3; THP-1, human monocytic leukemia cell line; SIRT1, Sirtuin 1; SOD, superoxide dismutase; Sox6, SRY-Box transcription factor 6.
